# Coronary Stent-Induced Kounis Syndrome: A Case Report

**DOI:** 10.7759/cureus.83208

**Published:** 2025-04-29

**Authors:** Takuya Kakumoto, Hirofumi Kawamata, Masatoshi Hori, Kuniyasu Harimoto, Tatsuya Kawasaki

**Affiliations:** 1 Department of Cardiology, Matsushita Memorial Hospital, Moriguchi, JPN; 2 Department of Emergency Medicine, Matsushita Memorial Hospital, Moriguchi, JPN

**Keywords:** coronary heart disease, kounis syndrome, metal allergy, stent, thrombosis

## Abstract

Kounis syndrome is an acute coronary syndrome triggered by allergic hypersensitivity to various stimuli. Here, we report the case of a 54-year-old man who had coronary stent-induced type III Kounis syndrome. The patient developed a rash on the anterior chest wall and experienced throat discomfort 40 minutes after stent implantation in the left anterior descending artery. Given his history of allergies, a presumptive diagnosis of anaphylaxis was made, and 0.5 mg of adrenaline was injected intramuscularly into the left thigh. The patient's symptoms initially resolved; however, approximately 50 minutes after the onset of the allergic reaction, he developed chest pain accompanied by ST-segment elevation in the anterior leads. Emergency coronary angiography revealed total occlusion at the mid-portion of the distal stent implanted in the left anterior descending artery, which was treated with repeated ballooning. The patient was later found to have a metal allergy. This case highlights the importance of obtaining a thorough allergy history, particularly regarding metal allergies, and recognizing Kounis syndrome as a potential cause of stent thrombosis associated with allergic hypersensitivity during coronary interventions.

## Introduction

Kounis syndrome is a rare but important clinical condition characterized by the occurrence of acute coronary events in the presence of an allergic reaction [1,2]. Although first described by Kounis and Zavras as histamine-induced coronary artery spasm [3], it has since been shown to cause not only coronary artery spasm but also atherosclerotic plaque rupture and thrombosis [4]. Currently, Kounis syndrome is classified into three types: coronary spasm without plaque erosion or rupture (Type I), coronary spasm with plaque erosion or rupture (Type II), and stent thrombosis (Type III) [5]. The exact incidence of Kounis syndrome remains unknown due to its frequent oversight and rarity; however, it is crucial to recognize this condition as a potential cause of acute coronary syndrome, as its management may differ from that of other etiologies [1,2]. Here, we report a case of coronary artery disease complicated by a metal allergy, in which allergic hypersensitivity developed following stent implantation, leading to stent thrombosis (Type III Kounis syndrome).

## Case presentation

**Figure 1:**
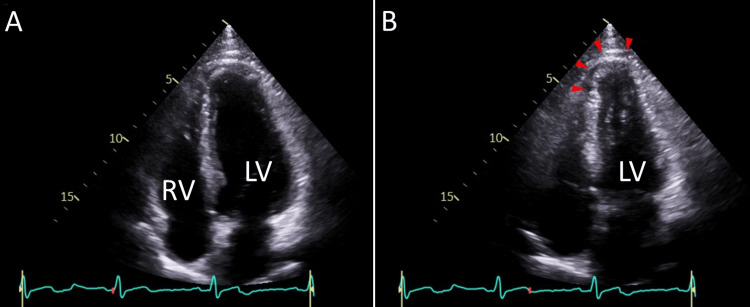
Echocardiography Echocardiographic apical four-chamber views at end-diastole (A) and end-systole (B) show mildly reduced wall motion in the anteroseptal wall and apex of the left ventricle (LV) (arrowheads). RV denotes the right ventricle.

**Figure 2:**
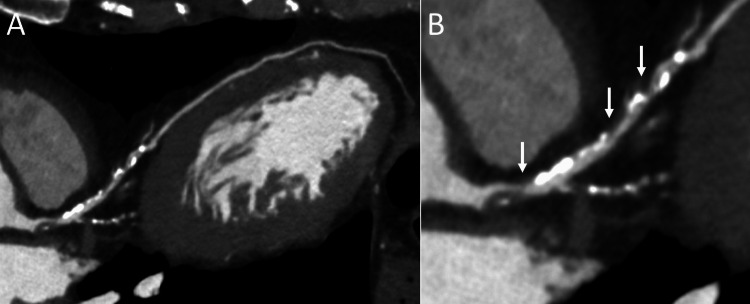
Coronary computed tomography angiography Computed tomography imaging reveals severe coronary stenosis in the left anterior descending artery (A) and an enlarged image of the same artery (B, arrows).

A 54-year-old man presented with a two-week history of chest discomfort, which was exacerbated by physical exertion. His vital signs and physical examination were normal. Electrocardiography, chest radiography, and laboratory tests were unremarkable. Echocardiography revealed mild hypokinesis in the anteroseptal wall and apex of the left ventricle, with a left ventricular ejection fraction of 62% (Figures 1A, 1B). A coronary computed tomography angiogram revealed severe coronary stenosis in the left anterior descending artery (Figures 2A, 2B). The patient was admitted for coronary intervention due to drug-refractory symptoms. He had a history of hypertension, dyslipidemia, and allergies to dust, cats, pollen, and other triggers, with varying reactions but no history of anaphylactic shock. His current medications included amlodipine (5 mg daily), rosuvastatin (5 mg daily), aspirin (100 mg daily), clopidogrel (75 mg daily), lansoprazole (10 mg daily), and bilastine (20 mg daily). He had quit smoking 28 years earlier after an eight-pack-year history and consumed alcohol in moderation. His father had a myocardial infarction at the age of 50 years.

**Figure 3:**
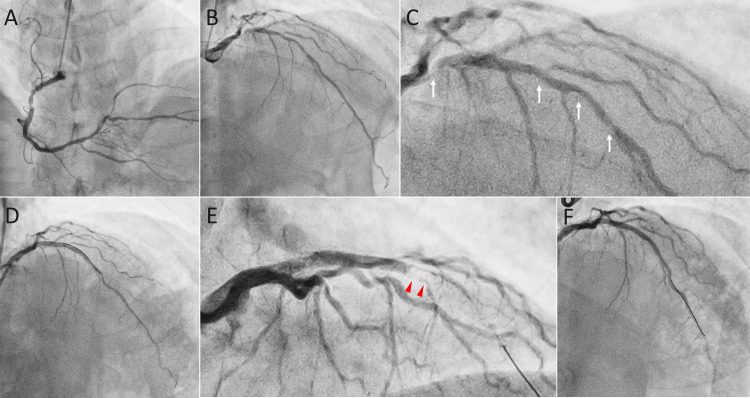
Coronary angiography The angiograms show mild stenosis in the right coronary artery (A) and moderate to severe stenosis in the left anterior descending artery (B and C, arrows). After stent implantation, successful coronary dilation was achieved (D), but stent thrombosis later developed in the distal portion of the stent (E, arrowheads). Stent patency was ultimately restored through repeated balloon angioplasty (F).

**Figure 4:**
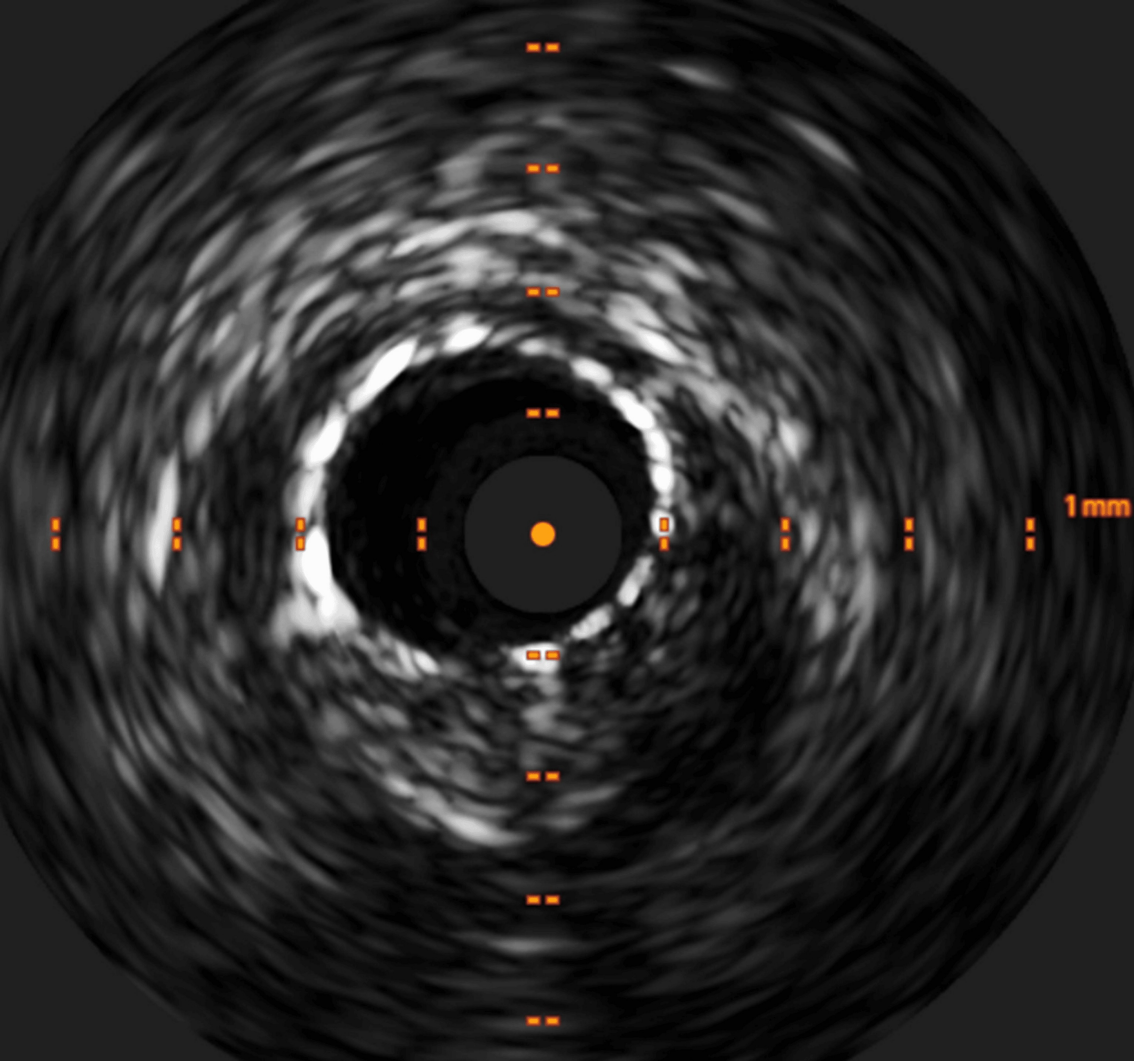
Intravascular ultrasound Optimal stent expansion and adequate stent strut apposition are demonstrated on ultrasound.

Coronary angiography, following the administration of 2,000 units of intravenous unfractionated heparin, revealed moderate to severe stenosis in the left anterior descending artery (Figures 3A-3C). The physiological assessment using the instantaneous wave-free ratio showed a value of 0.84, which gradually improved as the wire was withdrawn, indicating significant stenosis [6]. The lesion was initially dilated with balloons after an additional 5,000 units of intravenous unfractionated heparin. However, due to suboptimal results, stent implantation was performed. Approximately 40 minutes after stent implantation, the patient developed a rash on the anterior chest wall and experienced throat discomfort despite no new medications being administered after the procedure. The blood pressure was 137/93 mmHg, the heart rate was 60 beats per minute, and the oxygen saturation was 100% on ambient air. Given his allergic symptoms, a presumptive diagnosis of anaphylaxis of unknown cause was made. Adrenaline (0.5 mg) was injected intramuscularly into the left thigh, followed by intravenous dexamethasone sodium phosphate (6.6 mg). The activated clotting time was 311 seconds, and no ST-T segment changes were noted. Five minutes after the adrenaline injection, the patient's symptoms had almost completely resolved. Angiography and intravascular ultrasound confirmed stent patency (Figures 3D, 4), and the procedure was completed with post-stent dilation.

Approximately 50 minutes after the onset of the allergic reaction, the patient developed chest pain, accompanied by ST-segment elevation in the anterior leads. Emergency coronary angiography revealed total occlusion in the mid portion of the distal stent implanted in the left anterior descending artery (Figure 3E). The blood pressure was 90/43 mmHg, the heart rate was 73 beats per minute, and the oxygen saturation was 100% on ambient air. Intravenous noradrenaline was administered, and argatroban, a direct thrombin inhibitor with no cross-reactivity to heparin, was initiated due to the possibility of heparin-induced thrombocytopenia (HIT), given that the patient had previously received unfractionated heparin. Recanalization of the left anterior descending artery was achieved after repeated ballooning (Figure 3F), and excellent stent expansion was confirmed with intravascular ultrasound. Noradrenaline was discontinued, but intravenous argatroban and dexamethasone sodium phosphate were continued until patency of the left anterior descending artery was confirmed on angiography the following day.

The clinical course was uneventful, with the peak creatine kinase level reaching 2,892 U/L (reference range, 59 to 248), measured approximately 24 hours after the stent thrombosis. Further history revealed that the patient had a metal allergy, including silver, which resulted in a skin eruption. It was later confirmed that the antibody level for HIT was slightly elevated at 1.2 U/mL (reference range: <1.0), although platelet counts remained stable after the stent thrombosis (167,000 to 204,000 per microliter) compared to the pre-angiography count of 197,000 per microliter (reference range, 158,000 to 348,000). Neither the tryptase nor the IgE value was measured during the acute phase. The patient was discharged with instructions to avoid metal materials. He has been doing well without allergies or chest pain for more than six months after discharge.

## Discussion

The patient underwent coronary intervention for exertional angina, and allergic hypersensitivity developed after stent implantation. A presumptive diagnosis of anaphylaxis was made, and adrenaline was administered intramuscularly, followed by dexamethasone sodium phosphate. Although his symptoms were resolved, stent thrombosis occurred despite adequate stent dilation and appropriate procedural medications (i.e., dual antiplatelet therapy and prolonged activated clotting time). Stent thrombosis was treated with balloon angioplasty, along with intravenous noradrenaline and argatroban. The clinical course remained uneventful, although acute myocardial infarction ultimately occurred.

The exact trigger for the allergic hypersensitivity and the mechanism behind the stent thrombosis in the present patient remains unclear. It is well known that HIT is a serious complication of heparin use, which can lead to stent thrombosis during coronary intervention [7]. Diagnosing HIT is challenging, and its possibility as the cause of stent thrombosis in this patient cannot be completely excluded. Although there is no definitive diagnostic test for this condition, the 4Ts score is commonly used to assess the likelihood of HIT [8]. In this patient, the 4Ts score was 3, indicating a low probability of HIT [8]. Furthermore, the rash on the anterior chest wall and throat discomfort made a diagnosis of HIT unlikely, as thrombosis typically occurs without such allergic hypersensitivity in most patients with HIT.

Given his allergic history, including allergies to various triggers such as metals like silver, we can reasonably conclude that anaphylactic stent thrombosis, or type III Kounis syndrome, provoked by stent implantation, was the most likely diagnosis in this patient, although other causes of acute stent thrombosis, including mechanical issues, procedural complications, or high platelet reactivity, cannot be completely ruled out due to the lack of data, such as histological findings. Allergy tests could have confirmed our suspicion, but they were not performed due to ethical concerns, as they would have posed a risk of provoking stent thrombosis. This case underscores the importance of identifying metal allergies in patients scheduled for coronary intervention to help prevent coronary stent-induced allergic stent thrombosis, or type III Kounis syndrome, as demonstrated in this patient. Unfortunately, his metal allergy was not known prior to the procedure.

Adrenaline is well-established as the first-line treatment for anaphylactic shock. However, caution is warranted in patients with or suspected of having Kounis syndrome due to its involvement in the coronary arteries [9]. While experimental studies on the effects of alpha-2 adrenoceptors on regional coronary blood flow in both normal and ischemic myocardium are controversial, it has been reported that adrenaline may cause coronary vasoconstriction and increase oxygen demand [10,11]. Although no angiographic effects of adrenaline on the coronary arteries were observed in this patient, its administration should still be approached with caution in this context.

The pathophysiology of stent thrombosis in patients with Kounis syndrome appears to be multifactorial, with infiltration of eosinophils and mast cells into the coronary intima, media, and adventitia adjacent to the stent proposed as a key mechanism [5,12]. Although histological examination of the intima, media, and adventitia of the coronary artery was not performed, the procedure was carried out with adequate antiplatelet therapy and heparin administration. Furthermore, both angiography and intravascular ultrasound showed no evidence of coronary dissection, stent malapposition, or under-expansion, which supports Kounis syndrome as the likely cause of stent thrombosis.

## Conclusions

Our case highlights the importance of taking a thorough allergy history, particularly regarding metal allergies and recognizing Kounis syndrome as a potential cause of stent thrombosis associated with allergic hypersensitivity during coronary intervention. This is crucial, as the treatment for stent thrombosis caused by conditions like HIT may differ from that for stent thrombosis due to Kounis syndrome (i.e., anticoagulation vs. allergy suppression). Delayed recognition (e.g., recurrent thrombosis, and inappropriate treatment such as continued heparin use) could lead to fatal consequences.

## References

[1] Kounis NG (2016). Kounis syndrome: an update on epidemiology, pathogenesis, diagnosis and therapeutic management. Clin Chem Lab Med.

[2] Abdelghany M, Subedi R, Shah S, Kozman H (2017). Kounis syndrome: A review article on epidemiology, diagnostic findings, management and complications of allergic acute coronary syndrome. Int J Cardiol.

[3] Kounis NG, Zavras GM (1991). Histamine-induced coronary artery spasm: the concept of allergic angina. Br J Clin Pract.

[4] Douedi S, Odak M, Mararenko A (2023). Kounis syndrome: a review of an uncommon cause of acute coronary syndrome. Cardiol Rev.

[5] Biteker M (2010). A new classification of Kounis syndrome. Int J Cardiol.

[6] Davies JE, Sen S, Dehbi HM (2017). Use of the Instantaneous Wave-free Ratio or Fractional Flow Reserve in PCI. N Engl J Med.

[7] Al-Lamee RK, Gerber RT, Kooner JS (2010). Heparin-induced thrombocytopenia (HIT) as an unusual cause of acute stent thrombosis. Eur Heart J.

[8] Hogan M, Berger JS (2020). Heparin-induced thrombocytopenia (HIT): Review of incidence, diagnosis, and management. Vasc Med.

[9] Watanabe S, Sakai C, Hori M, Kawasaki T (2020). Insight into the time course of type III Kounis syndrome: a case report. J Emerg Med.

[10] Indolfi C, Piscione F, Villari B (1992). Role of alpha 2-adrenoceptors in normal and atherosclerotic human coronary circulation. Circulation.

[11] Overgaard CB, Dzavík V (2008). Inotropes and vasopressors: review of physiology and clinical use in cardiovascular disease. Circulation.

[12] Kounis NG, Koniari I, Roumeliotis A (2017). Thrombotic responses to coronary stents, bioresorbable scaffolds and the Kounis hypersensitivity-associated acute thrombotic syndrome. J Thorac Dis.

